# Crystal structure of 9-methacryloylanthracene

**DOI:** 10.1107/S2056989015004090

**Published:** 2015-03-11

**Authors:** Aditya Agrahari, Patrick O. Wagers, Steven M. Schildcrout, John Masnovi, Wiley J. Youngs

**Affiliations:** a Department of Chemistry, Cleveland State University, Cleveland OH 44115, USA; b Department of Chemistry, University of Akron, Akron OH 44325, USA; c Department of Chemistry, Youngstown State University, Youngstown OH 44555, USA

**Keywords:** crystal structure, aldol, methyl­ation, acetyl­anthracene, stereochemistry

## Abstract

In the title compound, C_18_H_14_O, with systematic name 1-(anthracen-9-yl)-2-methyl­prop-2-en-1-one, the ketonic C atom lies 0.2030 (16) Å out of the anthryl-ring-system plane. The dihedral angle between the planes of the anthryl and methacryloyl moieties is 88.30 (3)° and the stereochemistry about the C*sp* ^2^—C*sp* ^2^ bond in the side chain is *transoid*. In the crystal, the end rings of the anthryl units in adjacent mol­ecules associate in parallel–planar orientations [shortest centroid–centroid distance = 3.6320 (7) Å]. A weak hydrogen bond is observed between an aromatic H atom and the O atom of a mol­ecule displaced by translation in the *a*-axis direction, forming sheets of parallel-planar anthryl groups packing in this direction.

## Abstract 2

In the title compound, the substituted aromatic C atom lies 0.2030 (16) Å out of the anthryl plane, which forms a dihedral angle 88.30 (3)° with the plane of the *transoid* methacryloyl moiety.

## Chemical Context   

Enolizable aldehydes react with formaldehyde in strong aqueous base to form polyols, whereas ketones usually react to form polyhy­droxy­ketones (Davidson & Bogert, 1935 ▸; Vik *et al.*, 1973 ▸; Weissermel & Arpe, 1997 ▸; Wittcoff *et al.*, 2013 ▸). Therefore, the observed methyl­ation of 9-acetyl­anthracene by formaldehyde with alcoholic potassium carbonate (see Scheme below) is remarkable in that the reaction occurs with weak base in a non-aqueous medium by reduction of formaldehyde to form the methyl group (Pande *et al.*, 1998 ▸). Consequently, we obtained an X-ray structure determination to confirm the identity of the isolated product, 9-methacryl­oylanthracene or 1-(9-anthr­yl)-2-methyl-2-propen-1-one.
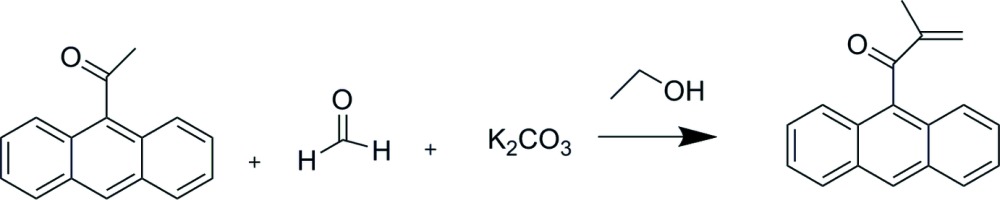


## Structural commentary   

**Figure 1:**
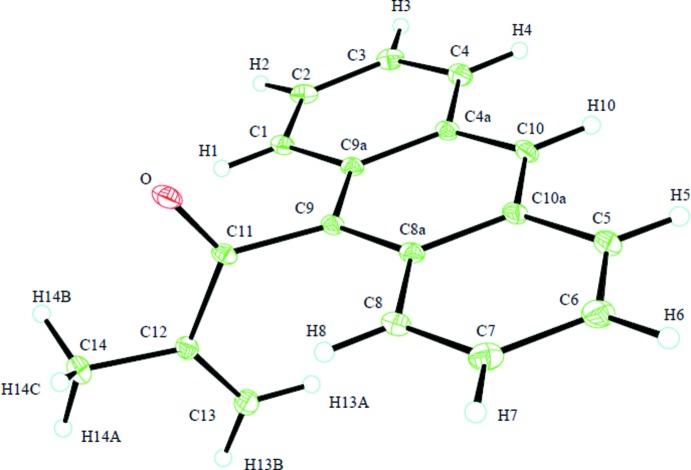
*ORTEP* (30% probability elipsoids) plot of the title compound showing the atom-labeling scheme.

The crystal structure (Fig. 1 ▸) establishes the material to be the α-methyl­ated aldol condensation product. Bond distances and valence angles agree well between the observed and the calculated structures. The anthryl ring system is essentially planar, as is the methacryloyl substituent (excepting the hydrogen atoms of the methyl group), whereas the calculated structure shows a slight deviation, about 7°, of the methacryl­oyl skeleton from planarity. The substituted C atom (C9) of the anthryl group also lies in the plane of the substit­uent, deviating by only 0.002 (2) Å. However, this C atom is puckered, so that the carbonyl C atom resides 0.2030 (16) Å out of the anthryl plane. This puckering is absent in the calculated structure. The planes of the anthryl and methacryloyl moieties are nearly perpendicular with a dihedral angle of 88.30 (3)° (but about 12° from perpendicular in the calculated structure). This general orientation is demanded by the close intra­molecular approach of the methacryloyl group to the *peri-*H atoms (H1 and H8), but packing effects may also contribute to deciding the exact angle since that calculated for the energy minimum differs by about 10° from that observed. The observed positioning is not quite symmetrical, with C11 being slightly closer (0.018 Å) to H1 than to H8. Similar geometries are found in 9-acetyl­anthracene, with a dihedral angle of 88.70 (3)° (Andersson *et al.*, 1984 ▸) and in 9-(bromo­acet­yl)anthracene, with a dihedral angle of 74.2 (1)° (Kubo *et al.*, 2007 ▸). Unfavorable non-bonded inter­actions in the present structure are likely the reason that the methyl group, which is bulkier than the methyl­ene group, projects away from the anthryl moiety, making the stereochemistry of the C11—C12 bond *transoid*. The puckering observed at C9 would partially relieve these unfavorable steric inter­actions occurring about this position.

## Supra­molecular_features   

**Figure 2:**
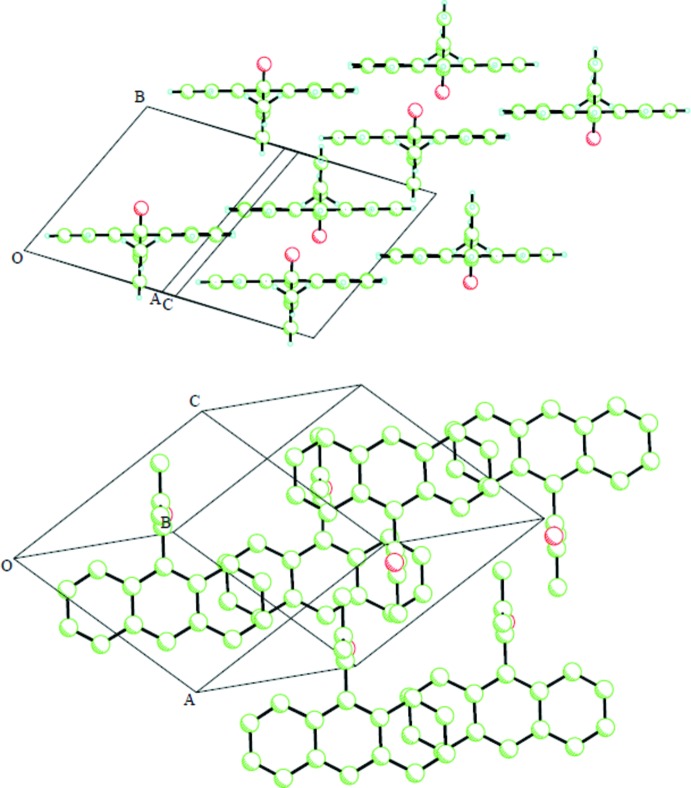
Views parallel to the planes of both the anthryl and the methacryloyl moieties (top) and parallel to the methacryloyl but perpendicular to the anthryl with H atoms omitted for clarity (bottom).

**Figure 3:**
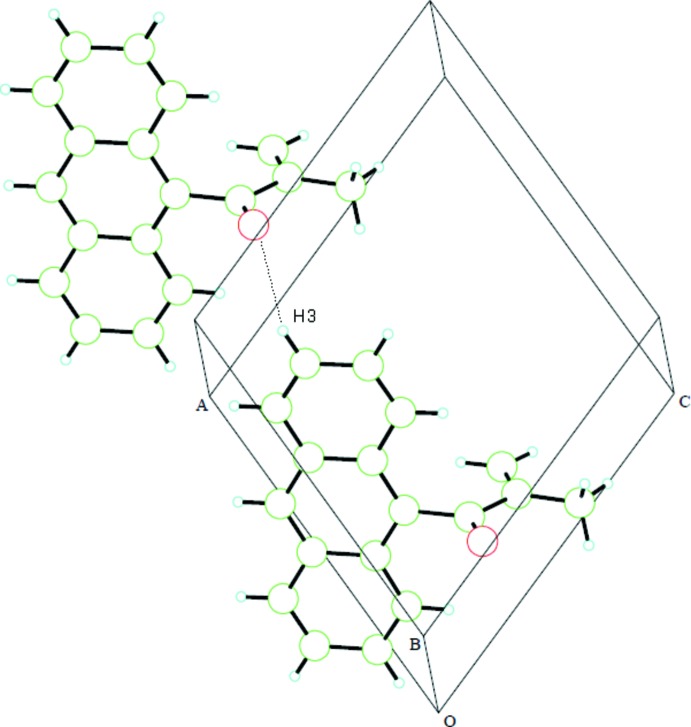
A fragment of a [100] hydrogen-bonded chain of mol­ecules in the crystal showing the intermolecular O⋯H close contact (dotted line).

Inter­molecular close contacts between large aromatic groups in the solid state often involve π–π stacking inter­actions involving parallel planar associations (Główka *et al.*, 1999 ▸). This motif is observed here as well, with the anthryl rings displaced and stacking alternately with those of neighboring mol­ecules (Fig. 2 ▸). The centroid–centroid separations are 3.6320 (7) and 3.7532 (7) and 3.7807 (8) Å. The methacryloyl substituent prevents such inter­actions involving the central ring of the anthryl moiety. A weak hydrogen bond is observed (Fig. 3 ▸) between an aromatic H atom (H3) and the O atom of a mol­ecule displaced by translation in the *a*-axis direction (Table 1 ▸), resulting in the formation of anthryl groups packing in parallel-planar sheets in this direction.

## Synthesis and crystallization   

Refluxing 9-acetyl­anthracene (1.0 g), paraformaldehyde (273 mg), and potassium carbonate (942 mg) in 3.0 ml ethanol afforded 80 mg product which eluted first from an alumina column with 10% ethyl acetate–hexane and was crystallized from chloro­form–hexane in the form of colorless plates.

## Refinement   

Crystal data, data collection and structure refinement details are summarized in Table 2 ▸. The H atoms were placed in calculated positions and refined as riding atoms, with C—H = 0.95 Å and *U*_iso_(H) = 1.2*U*_eq_(C-alkene and C-aromatic), and C—H = 0.98 Å and *U*_iso_(H) = 1.5 *U*_eq_(C-meth­yl).

## Calculations   

Density-functional theoretical computations were performed using *Gaussian* software (Frisch *et al.*, 2010 ▸) through the Ohio Supercomputing Center (in Columbus OH) with Zhao and Truhlar’s hybrid meta exchange-correlation functional, M06-2X, (Choe, 2012 ▸; Huh & Choe, 2010 ▸; Zhao & Truhlar, 2008 ▸), which is parameterized for non-metallic systems with non-covalent π–π interactions for accurate modelling of intramolecular dispersion effects. The basis set used is 6-31+G(d). To obtain the geometry at the global minimum potential energy, optimization was based on the minimum-energy conformation from a two-torsion MM2 plot (*ChemBio3D Ultra 12.0*; www.CambridgeSoft.com) using rotations about the C9—C11 and C11—C12 single bonds. The M06-2X structure has all vibrational frequencies positive, verifying that it is at a potential-energy minimum. Calculated values for geometrical paramters in the optimized isolated molecule are given in the Supporting information.

## Supplementary Material

Crystal structure: contains datablock(s) I. DOI: 10.1107/S2056989015004090/hb7286sup1.cif

Structure factors: contains datablock(s) I. DOI: 10.1107/S2056989015004090/hb7286Isup2.hkl

Supporting information file. DOI: 10.1107/S2056989015004090/hb7286Isup3.cml

CCDC reference: 1051418

Additional supporting information:  crystallographic information; 3D view; checkCIF report

## Figures and Tables

**Table 1:** Hydrogen-bond geometry (, )

*D*H*A*	*D*H	H*A*	*D* *A*	*D*H*A*
C3H3O^i^	0.95	2.48	3.3747(16)	157

Symmetry code: (i) 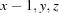.

**Table 2:** Experimental details

Crystal data	
Chemical formula	C_18_H_14_O
*M*_r_	246.29
Crystal system, space group	Triclinic, *P* 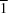
Temperature (K)	100
*a*, *b*, *c* ()	8.7602(5), 9.1784(5), 9.2032(5)
, , ()	67.206(2), 71.670(3), 75.195(2)
*V* (^3^)	639.98(6)
*Z*	2
Radiation type	Mo *K*
(mm^1^)	0.08
Crystal size (mm)	0.21 0.17 0.05

Data collection	
Diffractometer	Bruker APEXII CCD
Absorption correction	Multi-scan (*SADABS*; Bruker, 1997 ▸)
*T*_min_, *T*_max_	0.984, 0.996
No. of measured, independent and observed [*I* > 2(*I*)] reflections	12757, 2590, 2348
*R*_int_	0.026
(sin /)_max_ (^1^)	0.623

Refinement	
*R*[*F* ^2^ > 2(*F* ^2^)], *wR*(*F* ^2^), *S*	0.036, 0.106, 1.06
No. of reflections	2590
No. of parameters	173
H-atom treatment	H-atom parameters constrained
_max_, _min_ (e ^3^)	0.26, 0.21

Computer programs: *APEX2* and *SAINT* (Bruker, 1997 ▸), *SHELXS97* and *SHELXTL* (Sheldrick, 2008 ▸).

## supplementary crystallographic information

### Crystal data

**Table:**

C_18_H_14_O	*Z* = 2
*M**_r_* = 246.29	*F*(000) = 260
Triclinic, *P*1	*D*_x_ = 1.278 Mg m^−^^3^
Hall symbol: -P 1	Mo *K*α radiation, λ = 0.71073 Å
*a* = 8.7602 (5) Å	Cell parameters from 9953 reflections
*b* = 9.1784 (5) Å	θ = 2.5–28.4°
*c* = 9.2032 (5) Å	µ = 0.08 mm^−^^1^
α = 67.206 (2)°	*T* = 100 K
β = 71.670 (3)°	Plate, colourless
γ = 75.195 (2)°	0.21 × 0.17 × 0.05 mm
*V* = 639.98 (6) Å^3^	

### Data collection

**Table:**

Bruker APEXII CCD diffractometer	2590 independent reflections
Radiation source: fine-focus sealed tube	2348 reflections with *I* > 2σ(*I*)
Graphite monochromator	*R*_int_ = 0.026
φ and ω scans	θ_max_ = 26.3°, θ_min_ = 2.4°
Absorption correction: multi-scan (*SADABS*; Bruker, 1997)	*h* = −10→10
*T*_min_ = 0.984, *T*_max_ = 0.996	*k* = −11→11
12757 measured reflections	*l* = −11→11

### Refinement

**Table:**

Refinement on *F*^2^	Primary atom site location: structure-invariant direct methods
Least-squares matrix: full	Secondary atom site location: difference Fourier map
*R*[*F*^2^ > 2σ(*F*^2^)] = 0.036	Hydrogen site location: inferred from neighbouring sites
*wR*(*F*^2^) = 0.106	H-atom parameters constrained
*S* = 1.06	*w* = 1/[σ^2^(*F*_o_^2^) + (0.0479*P*)^2^ + 0.2652*P*] where *P* = (*F*_o_^2^ + 2*F*_c_^2^)/3
2590 reflections	(Δ/σ)_max_ < 0.001
173 parameters	Δρ_max_ = 0.26 e Å^−^^3^
0 restraints	Δρ_min_ = −0.21 e Å^−^^3^

### Special details

**Table:**

Geometry. All e.s.d.'s (except the e.s.d. in the dihedral angle between two l.s. planes) are estimated using the full covariance matrix. The cell e.s.d.'s are taken into account individually in the estimation of e.s.d.'s in distances, angles and torsion angles; correlations between e.s.d.'s in cell parameters are only used when they are defined by crystal symmetry. An approximate (isotropic) treatment of cell e.s.d.'s is used for estimating e.s.d.'s involving l.s. planes.
Refinement. Refinement of *F*^2^ against ALL reflections. The weighted *R*-factor *wR* and goodness of fit *S* are based on *F*^2^, conventional *R*-factors *R* are based on *F*, with *F* set to zero for negative *F*^2^. The threshold expression of *F*^2^ > σ(*F*^2^) is used only for calculating *R*-factors(gt) *etc*. and is not relevant to the choice of reflections for refinement. *R*-factors based on *F*^2^ are statistically about twice as large as those based on *F*, and *R*- factors based on ALL data will be even larger.

### Fractional atomic coordinates and isotropic or equivalent isotropic displacement parameters (Å^2^)

**Table:**

	*x*	*y*	*z*	*U*_iso_*/*U*_eq_	
O	0.88771 (10)	0.57102 (9)	0.67719 (10)	0.0221 (2)	
C1	0.49492 (14)	0.63847 (13)	0.64670 (14)	0.0176 (2)	
C2	0.35652 (15)	0.59642 (13)	0.64748 (15)	0.0203 (3)	
C3	0.21748 (14)	0.59128 (14)	0.77952 (15)	0.0214 (3)	
C4	0.22034 (14)	0.63126 (13)	0.90640 (14)	0.0202 (3)	
C4a	0.36214 (13)	0.67831 (13)	0.91022 (14)	0.0164 (2)	
C5	0.51116 (15)	0.80977 (14)	1.17279 (14)	0.0206 (3)	
C6	0.64792 (16)	0.85245 (14)	1.17433 (14)	0.0229 (3)	
C7	0.78992 (15)	0.85161 (14)	1.04616 (15)	0.0223 (3)	
C8	0.79096 (14)	0.80876 (13)	0.91974 (14)	0.0183 (3)	
C8a	0.64945 (13)	0.76406 (12)	0.91228 (13)	0.0153 (2)	
C9	0.64571 (13)	0.71974 (12)	0.78388 (13)	0.0148 (2)	
C9a	0.50386 (13)	0.67960 (12)	0.77832 (13)	0.0152 (2)	
C10	0.36743 (14)	0.71921 (13)	1.03939 (13)	0.0179 (3)	
C10a	0.50634 (14)	0.76402 (13)	1.04251 (13)	0.0166 (2)	
C11	0.80055 (13)	0.70042 (13)	0.65638 (13)	0.0150 (2)	
C12	0.84479 (14)	0.83810 (13)	0.50679 (13)	0.0178 (2)	
C13	0.74810 (16)	0.97827 (14)	0.48462 (15)	0.0242 (3)	
C14	1.00033 (15)	0.80523 (15)	0.38767 (15)	0.0264 (3)	
H1	0.5869	0.6405	0.5575	0.021*	
H2	0.3528	0.5701	0.5586	0.024*	
H3	0.1223	0.5599	0.7793	0.026*	
H4	0.1265	0.6278	0.9939	0.024*	
H5	0.4172	0.8103	1.2596	0.025*	
H6	0.6486	0.8830	1.2616	0.027*	
H7	0.8851	0.8812	1.0487	0.027*	
H8	0.8871	0.8086	0.8353	0.022*	
H10	0.2741	0.7164	1.1273	0.022*	
H13A	0.6505	0.9906	0.5644	0.029*	
H13B	0.7765	1.0662	0.3890	0.029*	
H14A	1.0155	0.9009	0.2910	0.040*	
H14B	0.9950	0.7164	0.3564	0.040*	
H14C	1.0920	0.7770	0.4379	0.040*	

### Atomic displacement parameters (Å^2^)

**Table:**

	*U*^11^	*U*^22^	*U*^33^	*U*^12^	*U*^13^	*U*^23^
O	0.0204 (4)	0.0189 (4)	0.0224 (4)	0.0011 (3)	−0.0021 (3)	−0.0070 (3)
C1	0.0187 (5)	0.0149 (5)	0.0175 (5)	−0.0013 (4)	−0.0045 (4)	−0.0042 (4)
C2	0.0244 (6)	0.0157 (5)	0.0228 (6)	−0.0011 (4)	−0.0109 (5)	−0.0057 (4)
C3	0.0177 (6)	0.0166 (5)	0.0285 (6)	−0.0039 (4)	−0.0105 (5)	−0.0015 (5)
C4	0.0148 (5)	0.0174 (5)	0.0218 (6)	−0.0025 (4)	−0.0032 (4)	−0.0007 (4)
C4a	0.0153 (5)	0.0118 (5)	0.0171 (5)	−0.0006 (4)	−0.0038 (4)	−0.0007 (4)
C5	0.0265 (6)	0.0168 (5)	0.0146 (5)	−0.0013 (5)	−0.0018 (5)	−0.0050 (4)
C6	0.0351 (7)	0.0191 (6)	0.0170 (6)	−0.0030 (5)	−0.0085 (5)	−0.0076 (5)
C7	0.0264 (6)	0.0193 (6)	0.0241 (6)	−0.0051 (5)	−0.0101 (5)	−0.0063 (5)
C8	0.0184 (6)	0.0168 (5)	0.0188 (6)	−0.0025 (4)	−0.0038 (4)	−0.0057 (4)
C8a	0.0173 (5)	0.0116 (5)	0.0149 (5)	−0.0007 (4)	−0.0040 (4)	−0.0030 (4)
C9	0.0158 (5)	0.0112 (5)	0.0141 (5)	−0.0009 (4)	−0.0030 (4)	−0.0018 (4)
C9a	0.0159 (5)	0.0109 (5)	0.0155 (5)	−0.0004 (4)	−0.0042 (4)	−0.0018 (4)
C10	0.0166 (5)	0.0153 (5)	0.0146 (5)	−0.0002 (4)	0.0004 (4)	−0.0021 (4)
C10a	0.0201 (6)	0.0122 (5)	0.0136 (5)	0.0000 (4)	−0.0031 (4)	−0.0025 (4)
C11	0.0146 (5)	0.0180 (5)	0.0155 (5)	−0.0037 (4)	−0.0041 (4)	−0.0076 (4)
C12	0.0193 (6)	0.0200 (6)	0.0154 (5)	−0.0071 (4)	−0.0017 (4)	−0.0068 (4)
C13	0.0299 (6)	0.0190 (6)	0.0189 (6)	−0.0054 (5)	−0.0016 (5)	−0.0037 (5)
C14	0.0249 (6)	0.0269 (6)	0.0215 (6)	−0.0077 (5)	0.0039 (5)	−0.0070 (5)

### Geometric parameters (Å, º)

**Table:**

O—C11	1.2176 (13)	C9—C8a	1.4024 (15)
C1—C2	1.3603 (17)	C9—C9a	1.4040 (16)
C1—H1	0.9500	C9—C11	1.5117 (14)
C2—C3	1.4201 (17)	C9a—C1	1.4300 (16)
C2—H2	0.9500	C9a—C4a	1.4365 (15)
C3—C4	1.3615 (17)	C10—C10a	1.3920 (17)
C3—H3	0.9500	C10—H10	0.9500
C4—C4a	1.4297 (16)	C10a—C5	1.4307 (16)
C4—H4	0.9500	C10a—C8a	1.4363 (15)
C4a—C10	1.3948 (16)	C11—C12	1.4864 (15)
C5—C6	1.3579 (18)	C12—C13	1.3277 (17)
C5—H5	0.9500	C12—C14	1.5020 (16)
C6—C7	1.4207 (17)	C13—H13A	0.9500
C6—H6	0.9500	C13—H13B	0.9500
C7—C8	1.3618 (16)	C14—H14A	0.9800
C7—H7	0.9500	C14—H14B	0.9800
C8—C8a	1.4301 (16)	C14—H14C	0.9800
C8—H8	0.9500		
			
C1—C9a—C4a	118.42 (10)	C9—C8a—C10a	119.11 (10)
C2—C1—C9a	120.88 (11)	C8—C8a—C10a	118.27 (10)
C2—C1—H1	119.6	C8a—C9—C9a	121.28 (10)
C9a—C1—H1	119.6	C8a—C9—C11	119.66 (10)
C1—C2—C3	120.84 (11)	C9a—C9—C11	118.84 (10)
C1—C2—H2	119.6	C9—C9a—C1	122.57 (10)
C3—C2—H2	119.6	C9—C9a—C4a	119.02 (10)
C4—C3—C2	120.12 (11)	C10a—C10—C4a	121.54 (10)
C4—C3—H3	119.9	C10a—C10—H10	119.2
C2—C3—H3	119.9	C4a—C10—H10	119.2
C3—C4—C4a	121.14 (11)	C10—C10a—C5	121.77 (10)
C3—C4—H4	119.4	C10—C10a—C8a	119.51 (10)
C4a—C4—H4	119.4	C5—C10a—C8a	118.72 (11)
C10—C4a—C4	121.93 (10)	O—C11—C12	120.54 (10)
C10—C4a—C9a	119.49 (10)	O—C11—C9	119.34 (10)
C4—C4a—C9a	118.57 (10)	C12—C11—C9	120.11 (9)
C6—C5—C10a	121.02 (11)	C13—C12—C11	120.05 (10)
C6—C5—H5	119.5	C13—C12—C14	124.29 (11)
C10a—C5—H5	119.5	C11—C12—C14	115.66 (10)
C5—C6—C7	120.35 (11)	C12—C13—H13A	120.0
C5—C6—H6	119.8	C12—C13—H13B	120.0
C7—C6—H6	119.8	H13A—C13—H13B	120.0
C8—C7—C6	120.66 (11)	C12—C14—H14A	109.5
C8—C7—H7	119.7	C12—C14—H14B	109.5
C6—C7—H7	119.7	H14A—C14—H14B	109.5
C7—C8—C8a	120.98 (11)	C12—C14—H14C	109.5
C7—C8—H8	119.5	H14A—C14—H14C	109.5
C8a—C8—H8	119.5	H14B—C14—H14C	109.5
C9—C8a—C8	122.61 (10)		
			
C9a—C1—C2—C3	−0.39 (17)	C9a—C9—C11—O	−85.73 (13)
C1—C2—C3—C4	1.10 (17)	C8a—C9—C11—C12	−91.47 (12)
C2—C3—C4—C4a	−0.25 (17)	C9a—C9—C11—C12	93.86 (12)
C3—C4—C4a—C10	−179.81 (10)	C9—C9a—C1—C2	178.37 (10)
C3—C4—C4a—C9a	−1.24 (16)	C4a—C9a—C1—C2	−1.12 (16)
C4—C4a—C10—C10a	179.63 (10)	C9—C9a—C4a—C10	0.99 (15)
C9a—C4a—C10—C10a	1.08 (16)	C1—C9a—C4a—C10	−179.50 (10)
C10a—C5—C6—C7	−0.34 (18)	C9—C9a—C4a—C4	−177.61 (9)
C5—C6—C7—C8	0.28 (18)	C1—C9a—C4a—C4	1.90 (15)
C6—C7—C8—C8a	0.20 (18)	C4a—C10—C10a—C5	178.48 (10)
C7—C8—C8a—C9	179.79 (10)	C4a—C10—C10a—C8a	−1.78 (16)
C7—C8—C8a—C10a	−0.58 (16)	C10—C10a—C5—C6	179.69 (10)
C9a—C9—C8a—C8	−178.68 (9)	C8a—C10a—C5—C6	−0.05 (17)
C11—C9—C8a—C8	6.78 (16)	C10—C10a—C8a—C9	0.39 (16)
C9a—C9—C8a—C10a	1.70 (16)	C5—C10a—C8a—C9	−179.85 (9)
C11—C9—C8a—C10a	−172.84 (9)	C10—C10a—C8a—C8	−179.25 (10)
C8a—C9—C9a—C1	178.13 (9)	C5—C10a—C8a—C8	0.51 (15)
C11—C9—C9a—C1	−7.29 (16)	O—C11—C12—C13	179.37 (11)
C8a—C9—C9a—C4a	−2.38 (16)	C9—C11—C12—C13	−0.21 (16)
C11—C9—C9a—C4a	172.20 (9)	O—C11—C12—C14	0.10 (15)
C8a—C9—C11—O	88.94 (13)	C9—C11—C12—C14	−179.48 (10)

### Hydrogen-bond geometry (Å, º)

**Table:**

*D*—H···*A*	*D*—H	H···*A*	*D*···*A*	*D*—H···*A*
C3—H3···O^i^	0.95	2.48	3.3747 (16)	157

Symmetry code: (i) *x*−1, *y*, *z*.

### Geometric parameters (Å, °)

Calculated values using RM06-2X/6-31+G(d) for optimized isolated molecule given in square brackets

**Table:**

O—C11	1.2176 (13)	[1.217]
C1—C2	1.3603 (17)	[1.365]
C1—H1	0.9500	[1.086]
C2—C3	1.4201 (17)	[1.428]
C2—H2	0.9500	[1.086]
C3—C4	1.3615 (17)	[1.364]
C3—H3	0.9500	[1.086]
C4—C4a	1.4297 (16)	[1.432]
C4—H4	0.9500	[1.087]
C4a—C10	1.3948 (16)	[1.396]
C5—C6	1.3579 (18)	[1.364]
C5—H5	0.9500	[1.087]
C6—C7	1.4207 (17)	[1.428]
C6—H6	0.9500	[1.086]
C7—C8	1.3618 (16)	[1.365]
C7—H7	0.9500	[1.086]
C8—C8a	1.4301 (16)	[1.434]
C8—H8	0.9500	[1.087]
C9—C8a	1.4024 (15)	[1.404]
C9—C9a	1.4040 (16)	[1.404]
C9—C11	1.5117 (14)	[1.509]
C9a—C1	1.4300 (16)	[1.434]
C9a—C4a	1.4365 (15)	[1.437]
C10—C10a	1.3920 (17)	[1.396]
C10—H10	0.9500	[1.089]
C10a—C5	1.4307 (16)	[1.432]
C10a—C8a	1.4363 (15)	[1.436]
C11—C12	1.4864 (15)	[1.497]
C12—C13	1.3277 (17)	[1.339]
C12—C14	1.5020 (16)	[1.502]
C13—H13A	0.9500	[1.087]
C13—H13B	0.9500	[1.087]
C14—H14A	0.9500	[1.093]
C14—H14B	0.9500	[1.095]
C14—H14C	0.9500	[1.095]
		
C2—C1—C9a	120.88 (11)	[120.8]
C2—C1—H1	119.6	[120.0]
C9a—C1—H1	119.6	[119.1]
C1—C2—C3	120.84 (11)	[120.8]
C1—C2—H2	119.6	[119.9]
C3—C2—H2	119.6	[119.4]
C2—C3—C4	120.12 (11)	[120.2]
C2—C3—H3	119.9	[119.5]
C4—C3—H3	119.9	[120.3]
C3—C4—C4a	121.14 (11)	[120.9]
C3—C4—H4	119.4	[120.8]
C4a—C4—H4	119.4	[118.3]
C10—C4a—C4	121.93 (10)	[121.6]
C10—C4a—C9a	119.49 (10)	[119.5]
C4—C4a—C9a	118.57 (10)	[119.0]
C6—C5—C10a	121.02 (11)	[120.9]
C6—C5—H5	119.5	[120.8]
C10a—C5—H5	119.5	[118.3]
C5—C6—C7	120.35 (11)	[120.2]
C5—C6—H6	119.8	[120.3]
C7—C6—H6	119.8	[119.5]
C6—C7—C8	120.66 (11)	[120.7]
C6—C7—H7	119.7	[119.4]
C8—C7—H7	119.7	[119.9]
C7—C8—C8a	120.98 (11)	[120.9]
C7—C8—H8	119.5	[119.9]
C8a—C8—H8	119.5	[119.2]
C8—C8a—C9	122.61 (10)	[122.5]
C8—C8a—C10a	118.27 (10)	[118.3]
C9—C8a—C10a	119.11 (10)	[119.2]
C8a—C9—C9a	121.28 (10)	[121.2]
C8a—C9—C11	119.66 (10)	[119.7]
C9a—C9—C11	118.84 (10)	[119.0]
C1—C9a—C4a	118.42 (10)	[118.4]
C1—C9a—C9	122.57 (10)	[122.5]
C4a—C9a—C9	119.02 (10)	[119.1]
C4a—C10—C10a	121.54 (10)	[121.5]
C4a—C10—H10	119.2	[119.2]
C10a—C10—H10	119.2	[119.2]
C5—C10a—C10	121.77 (10)	[121.6]
C8a—C10a—C10	119.51 (10)	[119.4]
C5—C10a—C8a	118.72 (11)	[119.0]
O—C11—C9	119.34 (10)	[120.5]
O—C11—C12	120.54 (10)	[120.2]
C9—C11—C12	120.11 (9)	[119.3]
C11—C12—C13	120.05 (10)	[120.4]
C11—C12—C14	115.66 (10)	[115.4]
C13—C12—C14	124.29 (11)	[124.2]
C12—C13—H13A	120.0	[121.8]
C12—C13—H13B	120.0	[121.0]
H13A—C13—H13B	120.0	[117.2]
C12—C14—H14A	109.5	[110.9]
C12—C14—H14B	109.5	[110.6]
C12—C14—H14C	109.5	[110.6]
H14A—C14—H14B	109.5	[109.2]
H14A—C14—H14C	109.5	[109.1]
H14B—C14—H14C	109.5	[106.5]
		
C9a—C1—C2—C3	-0.39 (17)	[-0.1]
C1—C2—C3—C4	1.10 (17)	[-0.1]
C2—C3—C4—C4a	-0.25 (17)	[0.2]
C3—C4—C4a—C10	-179.81 (10)	[179.7]
C3—C4—C4a—C9a	-1.24 (16)	[-0.2]
C4—C4a—C10—C10a	179.63 (10)	[-179.9]
C9a—C4a—C10—C10a	1.08 (16)	[0.0]
C10a—C5—C6—C7	-0.34 (18)	[-0.2]
C5—C6—C7—C8	0.28 (18)	[0.3]
C6—C7—C8—C8a	0.20 (18)	[0.0]
C7—C8—C8a—C9	179.79 (10)	[-179.9]
C7—C8—C8a—C10a	-0.58 (16)	[-0.3]
C9a—C9—C8a—C8	-178.68 (9)	[179.0]
C11—C9—C8a—C8	6.78 (16)	[0.2]
C9a—C9—C8a—C10a	1.70 (16)	[-0.6]
C11—C9—C8a—C10a	-172.84 (9)	[-179.4]
C8a—C9—C9a—C1	178.13 (9)	[-179.8]
C11—C9—C9a—C1	-7.29 (16)	[-1.1]
C8a—C9—C9a—C4a	-2.38 (16)	[1.1]
C11—C9—C9a—C4a	172.20 (9)	[179.8]
C8a—C9—C11—O	88.94 (13)	[102.3]
C9a—C9—C11—O	-85.73 (13)	[-76.5]
C8a—C9—C11—C12	-91.47 (12)	[-78.8]
C9a—C9—C11—C12	93.86 (12)	[102.4]
C9—C9a—C1—C2	178.37 (10)	[-179.0]
C4a—C9a—C1—C2	-1.12 (16)	[0.1]
C9—C9a—C4a—C10	0.99 (15)	[-0.7]
C1—C9a—C4a—C10	-179.50 (10)	[-179.9]
C9—C9a—C4a—C4	-177.61 (9)	[179.1]
C1—C9a—C4a—C4	1.90 (15)	[0.0]
C4a—C10—C10a—C5	178.48 (10)	[-179.7]
C4a—C10—C10a—C8a	-1.78 (16)	[0.4]
C10—C10a—C5—C6	179.69 (10)	[180.0]
C8a—C10a—C5—C6	-0.05 (17)	[-0.1]
C10—C10a—C8a—C9	0.39 (16)	[-0.1]
C5—C10a—C8a—C9	-179.85 (9)	[180.0]
C10—C10a—C8a—C8	-179.25 (10)	[-179.7]
C5—C10a—C8a—C8	0.51 (15)	[0.4]
O—C11—C12—C13	179.37 (11)	[172.7]
C9—C11—C12—C13	-0.21 (16)	[-6.1]
O—C11—C12—C14	0.10 (15)	[-5.8]
C9—C11—C12—C14	-179.48 (10)	[175.3]
